# Characterizing complete mitochondrial genome of *Aquilegia amurensis* and its evolutionary implications

**DOI:** 10.1186/s12870-024-04844-9

**Published:** 2024-02-28

**Authors:** Luyuan Xu, Jinghan Wang, Tengjiao Zhang, Hongxing Xiao, Huaying Wang

**Affiliations:** Key Laboratory of Molecular Epigenetics of Ministry of Education, Northeast Normal University, Changchun, 130024 China

**Keywords:** *Aquilegia*, Mitochondrial genome, Selection pressure analysis, Phylogenetic analysis

## Abstract

**Background:**

*Aquilegia* is a model system for studying the evolution of adaptive radiation. However, very few studies have been conducted on the *Aquilegia* mitochondrial genome. Since mitochondria play a key role in plant adaptation to abiotic stress, analyzing the mitochondrial genome may provide a new perspective for understanding adaptive evolution.

**Results:**

The *Aquilegia amurensis* mitochondrial genome was characterized by a circular chromosome and two linear chromosomes, with a total length of 538,736 bp; the genes included 33 protein-coding genes, 24 transfer RNA (tRNA) genes and 3 ribosomal RNA (rRNA) genes. We subsequently conducted a phylogenetic analysis based on single nucleotide polymorphisms (SNPs) in the mitochondrial genomes of 18 *Aquilegia* species, which were roughly divided into two clades: the European-Asian clade and the North American clade. Moreover, the genes *mttB* and *rpl5* were shown to be positively selected in European-Asian species, and they may help European and Asian species adapt to environmental changes.

**Conclusions:**

In this study, we assembled and annotated the first mitochondrial genome of the adaptive evolution model plant *Aquilegia*. The subsequent analysis provided us with a basis for further molecular studies on *Aquilegia* mitochondrial genomes and valuable information on adaptive evolution in *Aquilegia*.

**Supplementary Information:**

The online version contains supplementary material available at 10.1186/s12870-024-04844-9.

## Background

Mitochondria are semiautonomous organelle that carry out respiratory metabolism and provides energy for life activities [1]. Compared with those in the chloroplast genome, in plant mitochondrial genomes, genome rearrangements, repeat sequence recombination, and gene integration/loss/transfer/duplication occur frequently, greatly affecting the normal function of mitochondrial genes [2]. The mitochondrial genome structure is often described as circular, but its real structure appears to be diverse, such as circular, linear, and complex branched [3]. Additionally, the sequence evolution of the plant mitochondrial genome has been very slow, and the nucleotide synonym substitution rate has ranged from several to tens of times lower than that of plant chloroplast and nuclear genomes [4]. With the rapid development of genome sequencing technologies and assembly methods, the whole mitochondrial genomes of many plants have been assembled [5, 6]. The mitochondrial genomes play a key role in the adaptive evolution of plants, especially in response to abiotic stresses. They are involved in energy production, metabolism, regulation of PCD, and ROS production [7]. For example, the mitochondrial genomes of high-altitude plants may have experienced adaptative shrinkage to better survive extreme environments on the plateau [5]; similarly, the mitochondrial genome of *Geoffroea decorticans* (Gillies ex Hook. & Arn.) Burkart plants likely underwent structural changes facilitating their adaptation to the extreme conditions in the Atacama Desert [8].

*Aquilegia* L. (columbine) is a perennial herb of the Ranunculaceae family [9]. Along with several new species being reported, approximately 110 species of columbine taxa have been found, which is an ideal material for studying adaptive radiation evolution [10]. The chromosomal-level genome and chloroplast genome of *Aquilegia* have previously been reported, but the phylogenetic relationships of *Aquilegia* based on different genomes are controversial, which have made the evolution of adaptive radiation in *Aquilegia* unclear. However, systematic research and evolutionary analysis of the *Aquilegia* mitochondrial genome are lacking [11, 12]. The assembly and analysis of the mitochondrial genome could provide a new perspective and reference for the identification of the phylogeny of *Aquilegia.* This approach can help us to realize and understand how the mitochondrial genome changed during the adaptive evolution of *Aquilegia*.

In this study, we assembled the complete mitochondrial genome of *Aquilegia amurensis* Kom. and analyzed codon usage bias to reveal characteristics of mitochondrial genome. In order to understand the phylogenetic relationships and adaptive evolution *A. amurensis* of *Aquilegia* from the mitochondrial genome perspective. First, we identified SNPs from 18 species including North American, European, and Asian species. Then, phylogenetic trees were constructed based on the SNPs to lay a foundation for inferring the evolutionary history of *Aquilegia*. Finally, we explored positive selection in *Aquilegia* to advance the current genetic and evolutionary understanding of this genus.

## Materials and methods

### Plant material and mitochondrial genome assembly

Fresh leaves of *A. amurensis* were collected from Changbai Mountain (N 42.053, E 128.048; Jilin Province, China). The identification of plants was conducted by Hongxing Xiao. The deposition number was 220730-0101 and the voucher number was NENU403102, these materials were deposited in the Northeast Normal University Herbarium in Changchun, China. High-quality DNA was extracted using the modified cetyltrimethylammonium bromide (CTAB) method [13], and evaluated using a Qubit 3.0 Fluorometer (Life Technologies, Carlsbad, CA, USA) and a NanoDrop One spectrophotometer (Thermo Fisher Scientific). PacBio HiFi sequencing was performed on the PacBio Sequel II platform (Pacific Biosciences, CA, USA) according to the manufacturer’s instructions.

The *de novo* assembly of mitochondrial genome was performed using Flye [14] based on the long subreads. In terms of parameter setting, the assembly was set four times in the minimum overlapping sequences of 1000, 3000, 5000 and 10,000, and the other parameters were set to their defaults. We used Bandage [15] to visualize the mitochondrial genome sketch. The mitochondrial sequences of *A. amurensis* were selected with BLASTn [16] using the complete mitochondrial sequences of *Liriodendron tulipifera* L. (NC_021152.1) as query. Due to the complexity of the sketch and the existence of some repeated sequences, BWA [17] was used to map the HiFi data to the graphical mitochondrial genome fragments, which excluded the repeated sequences to ensure that the assembly result was supported by a larger number of reads.

The mitochondrial genome of *A. amurensis* was initially annotated using GeSeq tools [18] on the MPI-MP CHLOROBX website (https://chlorobox.mpimp-golm.mpg.de) with the reference mitogenome (*L. tulipifera*, GenBank: NC_021152.1). The tRNA genes were identified using tRNAscan-SE (http://lowelab.ucsc.edu/tRNAscan-SE) [19], while the rRNA genes were annotated using BLASTn software. Apollo [20], an interactive tool that allows biological experts to improve these approximations by viewing and independently evaluating the data supporting each annotation, was used to manually correct annotation errors in each mitochondrial genome. The mitochondrial map was drawn using Organellar Genome DRAW (OGDRAW) [21].

### Codon usage bias and repeated sequence analysis

Phylosuite v1.1.16 [22] was used to extract the protein-coding sequences of the *A. amurensis* mitochondrial genome. MEGA v7.0.26 [23] was used to analyze basic sequence information such as base composition, nucleotide sequence information sites, start codons, and stop codons and to calculate relative synonymous codon usage (RSCU). The results were visualized using the R package ggplot2 [24].

Simple sequence repeat (SSRs), tandem repeats and dispersed repeats were detected using the MISA web server (https://webblast.ipk-gatersleben.de/misa/) [25], the Tandem Repeats Finder web server v4.09 (https://Tandem.bu.edu/trf/trf.unix.help.html/) [26] and the REPuter Network web server (https://bibiserv.cebitec.uni-bielefeld.de/reputer/) [27], respectively. The results were visualized using the R package ggplot2 [24]. In addition, we used ROUSFinder.py to analyze non-tandem repeats [28].

### Phylogenetic analysis

To investigate the phylogenetic relationships of *Aquilegia* species based on the mitochondrial genome, we collected whole-genome sequencing data from 18 *Aquilegia* species, including *A. sibirica* Lam., *A. ecalcarata* Maxim., and *A. amurensis*. *A. parviflora* Ledeb., *A. rockii* Munz, *A. viridiflora* Pall., *A. yabeana* Kitag., *A. oxysepala* var. *kansuensis* Brühl, *A. oxysepala* var. *oxysepala* Trautv. et Mey., *A. japonica* Nakai & Hara, *A. canadensis* L., *A. coerulea* E. James, *A. barnebyi* Munz, *A. longissimi* A. Gray, *A. chrysantha* A. Gray, *A. formosa* Fisch. ex DC, *A. aurea* Janka, *A. vulgaris* Richardson, from the National Center for Biotechnology Information (NCBI, http://www.ncbi.nlm.nih.gov/sra) (Table S1).

The raw data were processed in two steps: adapter sequences in the reads were trimmed and then reads that contained more than 50% low quality bases (quality value ≤ 5) were removed. The remaining sequencing reads from *Aquilegia* species were aligned separately to the mitochondrial genome of *A. amurensis* using BWA [17]. The readgroups were added using the AddOrReplaceReadGroups module in GATK v.4.1.8.0 [29], and the MarkDuplicates module was subseqently used to mark the duplicates. The index was subsequently built through SAMtools v.0.1.18 [30]. The SelectVariants module in GATK v.4.1.8.0 was used to extract the original SNPs. SNPs with mapping quality ≥ 40 and a SNP quality ≥ 30 were retained. VCFtools v0.1.13 [31] was used to remove SNPs with a minor allele frequency (MAF) of 0.05 or less, more than 2 alleles, a mean sequencing depth of less than 50, or missing rate of more than 0.5. Moreover, only homozygous SNPs were retained [32]. *Semiaquilegia adoxoides* Makino (SRR437677) and *Paraquilegia microphylla* Drumm. et Hutch (SRR26400723, unpublished) were considered outgroups [11, 33] (Table S1). The maximum likelihood (ML) method was used for phylogenetic analysis using IQTREE v2.2.2.6 with 1000 ultrafastbootstrap replicates [34]. According to the Bayesian information criterion (BIC), the best-fit model for the SNP datasets was the TVM + F + G4 model. Meanwhile, Bayesian inference (BI) method was used to reconstruct a phylogenetic tree using MrBayes v3.2 [35] under the GTR + G + I model. The Markov Chain Monte Carlo (MCMC) analyses were run for 1000,00 generations. The trees were sampled every 100 generations, and the first 25% of the trees were discarded as burn-in. The final trees were visualized using Figtree v1.4.4 (http://tree.bio.ed.ac.uk/software/figtree/) and iTOL v4.0 (https://itol.embl.de/itol.cgi). The phylogenetic trees of the different genome were compared using the R package phytools [36].

### Selection pressure analysis

Using the annotated information of *A. amurensis* as a reference and SNPs, we obtained the aligned CDS of the mitochondrial genomes. The nonsynonymous substitution rate (*d*_*N*_), synonymous substitution rate (*d*_*S*_), and *d*_*N*_*/d*_*S*_ ratio of each protein-coding gene were calculated by the yn00 module using the Codeml program in the PAML package [37]. The *d*_*N*_*/d*_*S*_ ratio is an important index of selection pressure. A *d*_*N*_*/d*_*S*_ ratio > 1, *d*_*N*_*/d*_*S*_ ratio = 1, and *d*_*N*_*/d*_*S*_ ratio < 1 indicated positive, neutral, and negative selection, respectively [38]. Moreover, the selection pressure of European species and Asian species, the selection pressure of North American species, and the selection pressure between the above two groups were compared. The results were visualized using the R package ggplot2 [24].

## Results

### Complete mitochondrial genome sequence of *A. amurensis*

A total of 22,851,573 subreads were obtained from the PacBio Sequel II platform and the N50 of the subreads was 20,484 bp. Based on long-read data from the PacBio Sequel platform, the main structure of the mitochondrial genome was multibranch structure. We obtained six nodes of the *A. amurensis* mitochondrial genome, which were named on each node and formed overlapping areas with each other along the connecting lines to form a complex genome structure (Fig. S1). Even contig4-contig5-contig6 and contig1-contig2-contig4 can form a closed ring structure, where contig2 and contig6 were not included. The above three different types of assembly results were all confirmed computationally (Fig. S1). To succinctly describe the mitochondrial genome and avoid redundancy of partial sequences, we used a circular ring structure (contig1-contig3-contig5-contig4) and two linear fragments (contig2 and contig6) as representative sequences of the mitochondrial genome.

**Fig. 1:**
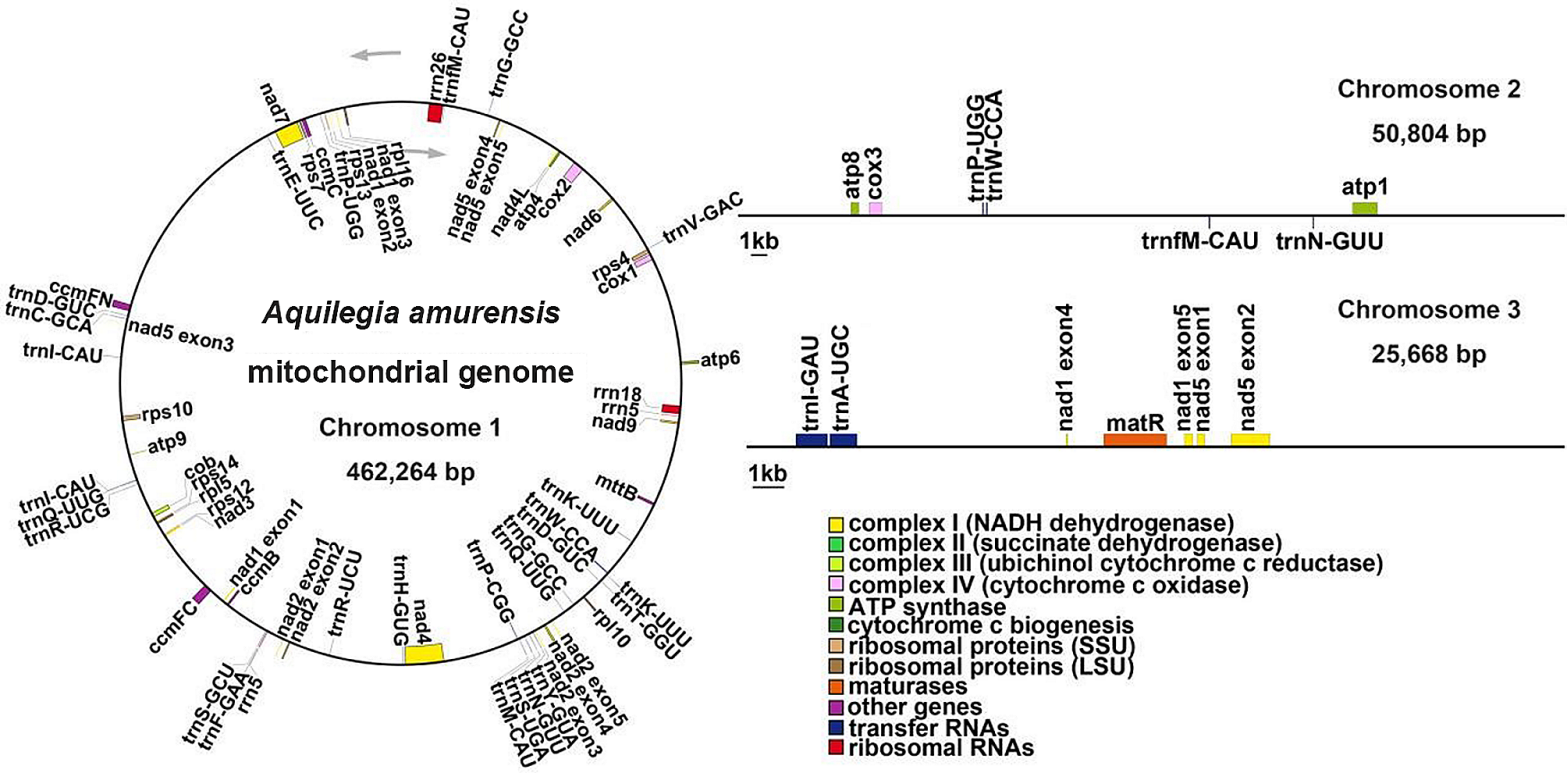
Schematic of the mitochondrial genome of *A. amurensis*. Genes belonging to different functional groups are color-coded

The total length of mitochondrial genome of *A. amurensis* was 538,736 bp, and the GC content was 46.21%. Chromosome 1 had a circular structure, a length of 462,264 bp, and a GC content of 46.22%. Chromosome 2 and 3 were linear fragments. The lengths of these fragments were 50,804 bp and 25,668 bp, respectively, and the GC content was 44.92% and 48.64%, respectively (Fig. 1). Moreover, a total of 24 tRNA genes, 3 rRNA genes and 33 unique protein-coding genes were annotated, including 24 unique mitochondrial core genes and 9 noncore genes (Table S2). There were five ATP synthase genes *(atp1*, *atp4*, *atp6*, *atp8* and *atp9*); nine NADH dehydrogenase genes (*nad1*, *nad2*, *nad3*, *nad4*, *nad4L*, *nad5*, *nad6*, *nad7*, and *nad9*); four cytochrome C biogenetic genes (*ccmB*, *ccmC*, *ccmFC* and *ccmFN*); three cytochrome C oxidase genes (*cox1*, *cox2* and *cox3*), one membrane transport-protein-gene (*mttB*); one mature enzyme-encoding gene (*matR*); and one panthenol-cytochrome C reductase gene (*cob*) annotated in the mitochondrial genome. The noncore genes included three ribosomal large subunit genes (*rpl5*, *rpl10*, and *rpl16*), six ribosomal small subunit genes (*rps4*, *rps7*, *rps10*, *rps12*, *rps13*, and *rps14*) (Fig. 1, Table S2).

### Relative synonymous codon usage of *A. amurensis* mitochondrial genome

**Fig. 2:**
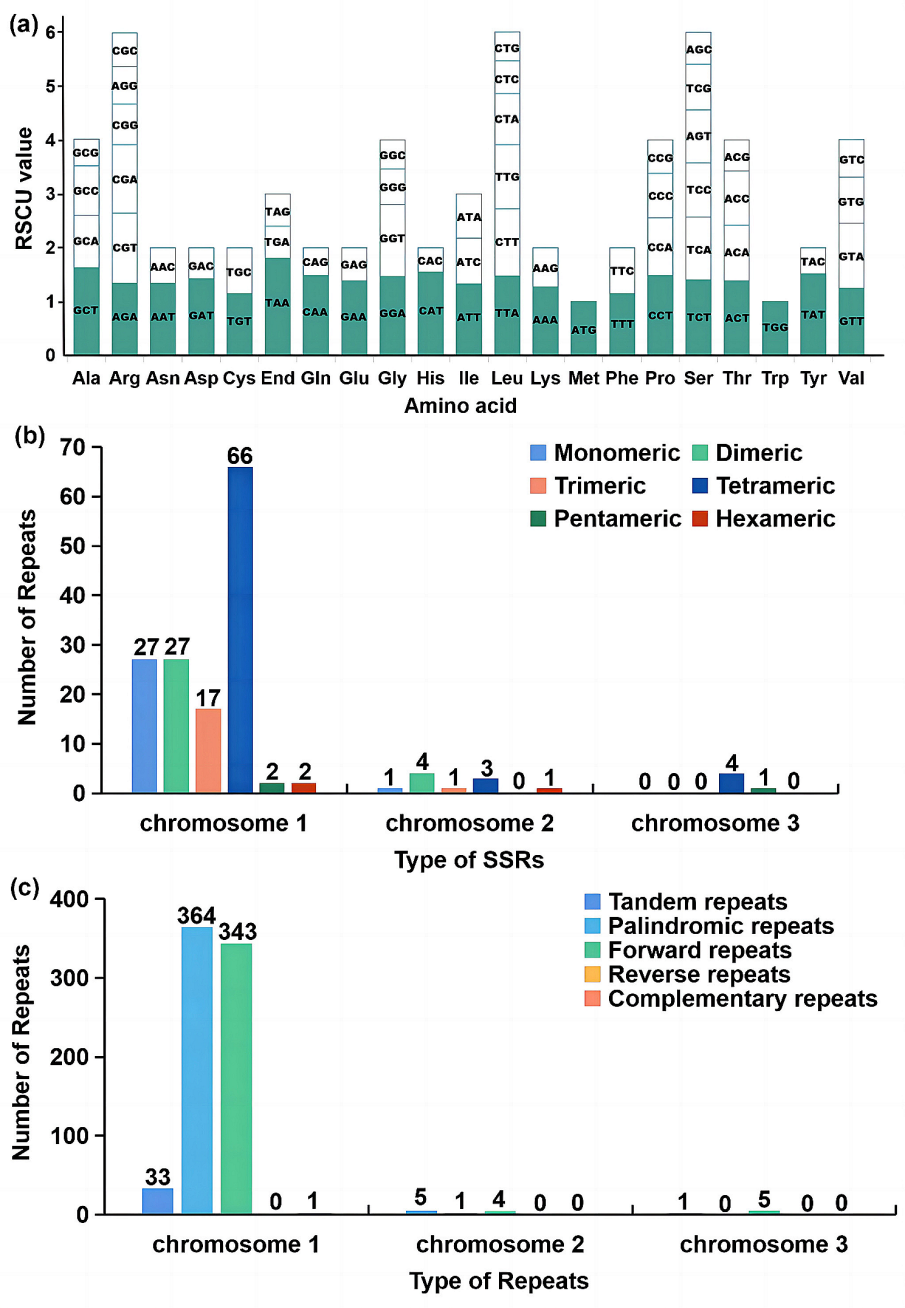
Relative synonymous codon usage (**a**), SSRs (**b**) and other repeats (**c**) in the mitochondrial genome of *A. amurensis*

Codon usage bias is thought to be the result of a relative balance within the cell over a long period of evolutionary selection. An RSCU value greater than 1 was considered to indicate the beneficial effect of amino acids. There were 27 codons for which RSCU > 1 (Fig. 2a). The RSCU values of the start codon AUG and tryptophan (UGG) were both 1. The remaining mitochondrial protein-coding genes exhibited general codon usage (Fig. 2a). The stop codon had a high preference for UAA, and its RSCU value was 1.8, which was the highest among the mitochondrial protein-coding genes. Alanine (Ala) had the second highest preference for GCU codons, with the an RSCU value of 1.61(Fig. 2a). Notably, cysteine (Cys) and phenylalanine (Phe) did not have strong codon usage with maximum RSCU values less than 1.2 (Fig. 2a). The results showed that A or T nucleotides were used more frequently at the third codon position than were C or G nucleotides.

### Repeat sequence analysis of the *A. amurensis* mitochondrial genome

In total, we detected 156 SSRs, including 141 SSRs on chromosome 1, 10 SSRs on chromosome 2 and 5 SSRs on chromosome 3. In addition to chromosome 3, monomeric and dimeric nucleotide repeats on chromosomes 1 and 2, which contained 54 (38.30%) and 5 (50.00%), respectively (Table S3). There were 39 tandem repeats in the mitochondrial genome with more than 73% match and a length between 12 and 72 bp (Table S4). For nontandem repeats, 718 pairs of other repeats with a length greater than or equal to 30 bp were found, including 365 pairs of palindromic repeats, 352 pairs of forward repeats, and 1 pair of complementary repeats; moreover, no reverse repeats or long repeats (> 1000 bp) were found.

On chromosome 1, adenine (A) monomeric repeats accounted for 62.96% (17) of the twenty-seven monomeric SSRs (Fig. 2b). TA and CT repeats were the most common types of dimeric SSRs, accounting for 51.85% of the dimeric SSRs (Table S3). On chromosome 1, there were two hexametric SSRs. Thirty-three tandem repeats were matching degree greater than 72% and length ranging from 13 to 72 bp (Table S4). A total of 708 dispersed repeats with lengths greater than or equal to 30 were observed, including 364 palindromic repeats, 343 forward repeats and 1 complementary repeat, and no reverse repeats were detected (Fig. 2c). The longest palindromic repeat was 310 bp and the longest forward repeat was 231 bp (Table S5). On chromosome 2 and 3, there were few SSRs, tandem repeats or dispersed repeats. Moreover, reverse repeats or complementary repeats were not found.

### Phylogenetic analysis of *Aquilegia*

**Fig. 3:**
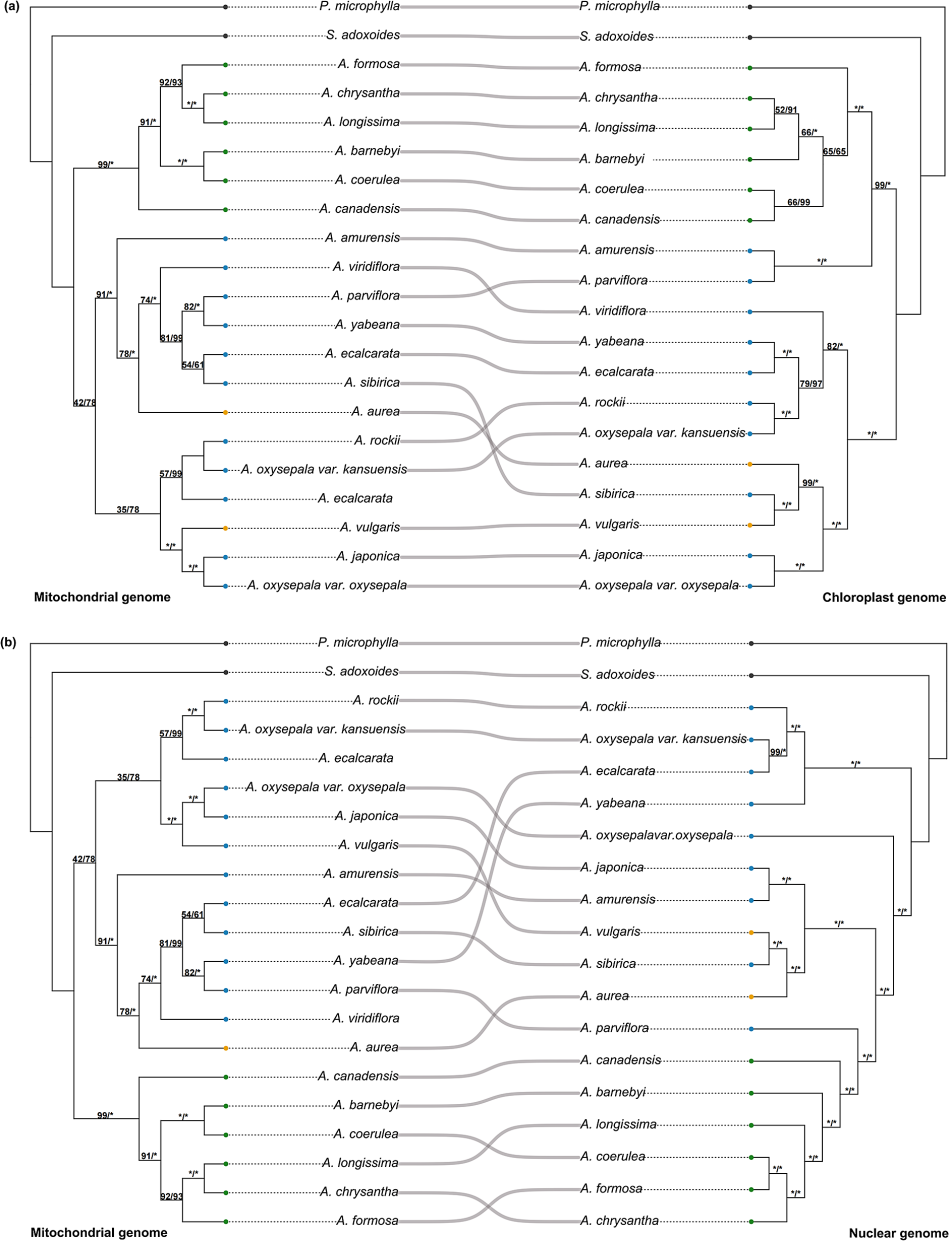
Comparison of phylogenetic relationship in *Aquilegia*. Comparison of phylogenetic tree between mitochondrial and chloroplast genome (**a**); comparison of phylogenetic relationship between mitochondrial and nuclear genome (**b**). The phylogenetic trees of mitochondrial genome, chloroplast genome and nuclear genome were constructed based on 1533, 599 and 363,842 SNPs, respectively. The blue, orange, and green dots represent species from Asia, Europe, and North America, respectively. The gray dots represent outgroup species. The ML ultrafastbootstrap (ufbs) and BI posterior probability (PP) values are indicated above the branches. “*” are ufbs or PP of 100

To explore the phylogenetic relationships between *Aquilegia* species and to clarify the phylogenetic position of *A. amurensis*, trees were constructed using 1533 SNPs of mitochondrial genome and 97 SNPs located in genes. The ML tree and Bayesian tree showed the same topology, and the posterior probabilities of the Bayesian tree for each lineage were greater (Fig. S2). Based on the phylogenetic tree of mitochondrial genome, approximately two clades were identified within *Aquilegia*. One clade included all the species from Asia and Europe, while the other clade included six species distributed in North America. Despite the low ultrafastbootstrap (ufbs value = 42) and posterior probability (PP value = 78) of the European-Asian clade, there were strong supports for the European-Asian clade in the phylogenetic trees based on chloroplast and nuclear genomes data (Fig. 3). The clade containing European and Asian species was divided into two subclades. *A. amurensis* and *A. parviflora* were in the same subclade, while the topology supported *A. japonica* and *A. oxysepala* var. *oxysepala* as sister branches, and *A. vulgari*s from Europe had a common ancestor. *A. ecalcarata* from the eastern group and the western group were divided into two subclades. *A. ecalcarata* from the eastern group was closely related to *A.sibirica*, and *A. ecalcarata* from the western group formed a single clade with *A. rockii* and *A. oxysepala* var. *kansuensis* (Fig. S2).

### Selection pressure analysis of *Aquilegia* mitochondrial protein-coding genes

**Fig. 4:**
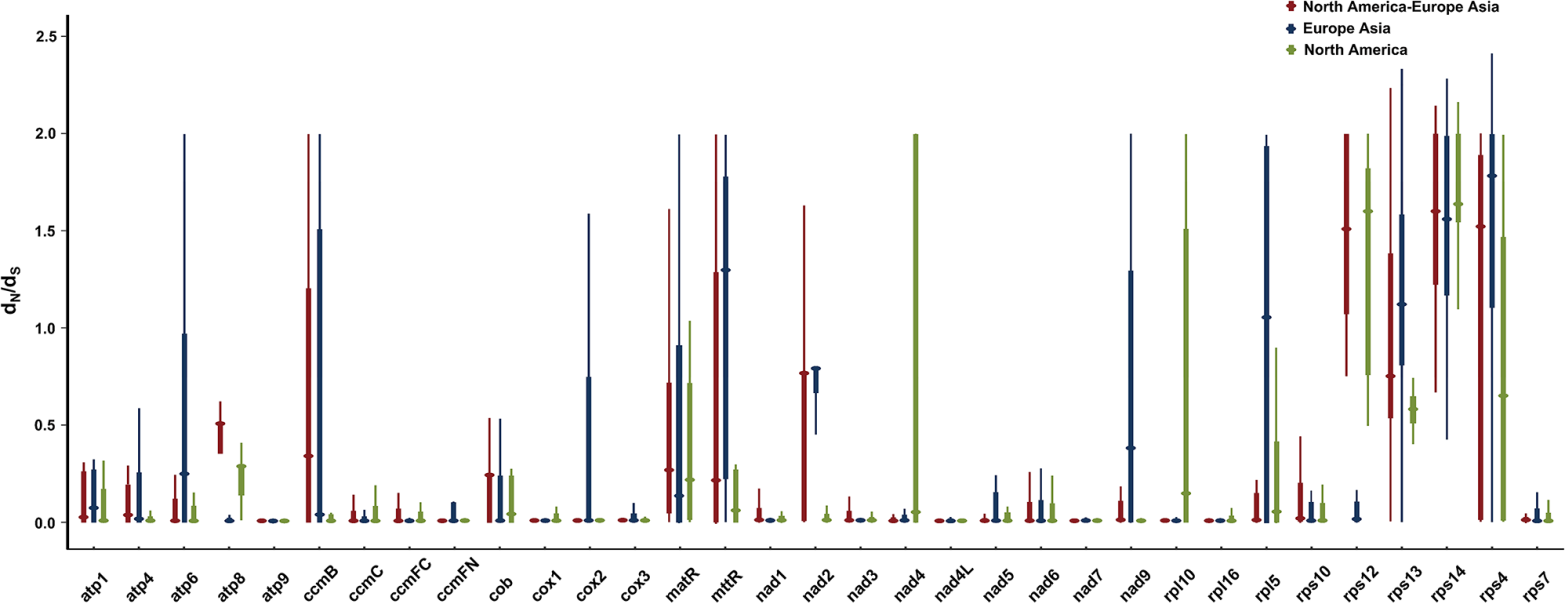
*d*_*N*_*/d*_*S*_ ratios of protein-coding genes in the mitochondrial genome of *Aquilegia*. The upper and lower limits and circles in the thick lines represent the upper quartile, lower quartile, and median of the pairwise *d*_*N*_*/d*_*S*_ ratios of each gene, respectively

It is important to determine the nonsynonymous substitution rate (*d*_*N*_*)* and synonymous substitution rate (*d*_*S*_) as these parameters are highly important for understanding the evolutionary dynamics of protein-coding sequences. The *rps14* gene was positively selected, as indicated by a *d*_*N*_*/d*_*S*_ ratio greater than 1.0 in all three comparisons. (Table S6). In addition, the genes *mttB*, *rpl5*, *rps4*, and *rps13* were positively selected in European and Asian species (Fig. 4). According to the comparison between European-Asian species and North American species, the genes *rps4* and *rps12* were also under positive selection (Fig. 4). Compared with those of other protein-coding genes, the genes *atp9*, *ccmC*, *ccmFC*, *cox1*, *cox3*, *nad1*, *nad3*, *nad4L*, *nad6*, *nad7*, *rpl16*, and *rps7* had significantly lower *d*_*N*_*/d*_*S*_ ratios, indicating that their functions were highly conserved (Table S6).

## Discussion

### Characterization of the *A. amurensis* mitochondrial genome

The mitochondrion is a semiautonomous organelle with its own genetic material and genetic system, and it has the ability to independently replicate and inherit [1]. The best-known function of mitochondria is the conversion of proton concentration gradients to ATP for biological activity through oxidative phosphorylation. Because of the energy metabolism of mitochondrial DNA, its role in adaptive evolution has attracted much attention. In particular, during evolution, mitochondria play a crucial role in the internal regulation of organisms under harmful environmental conditions such as hypoxia and low temperature [39]. Here, we sequenced, assembled, and analyzed the mitochondrial genome of *A. amurensis* to understand how *Aquilegia* adaptively evolved from the perspective of the mitochondrial genome. After the repeat regions were excluded from the HiFi data, we obtained one circular contig and two linear contigs.

Changes in the size and structure of plant mitochondrial genomes are obvious, but functional genes remain conserved [40]. The mitochondrial genome of the common ancestor of angiosperms consists of 41 protein-coding genes [41]. Approximately 33 out of the 41 protein-coding genes were detected in the *Aquilegia* mitochondrial genome. Compared with those in *A. thaliana*, the genes *rpl2*, *rps3*, and *rps7* were missing. The total length of mitochondrial genome of *Aquilegia* was greater than that of *Arabidopsis thaliana* L., *Oryza sativa* L., *Triticum aestivum* L. and other model plants [42–44], but lower than that of *Zea mays* L [45]. There were fewer genes in *A. amurensis* than in the above model plants. The transfer of mitochondrial genes to the nuclear genome may explain the above results, meaning that gene transfer between the cell nucleus and cytoplasm may be more frequent in *A. amurensis*. Gene transfer from mitochondrial to the nuclear genome has been common during plant evolution [46].

Mitochondria and chloroplast are self-renewing organelles with independent genomes. The total length of the mitochondrial genome was 538 kb, which was three times that of the chloroplast genome. However, the gene density of the mitochondrial genome was one-third that of chloroplast genome [12]. The GC content of the chloroplast genome is often lower than that of the mitochondrial genome [6].

The RSCU is closely related to the evolution of organisms. On the one hand, codon preference can affect amino acid sequences, protein structure and function, thus affecting the adaptability and survival ability of organisms. On the other hand, codon preference can also reflect the evolutionary process and trend of the genome. The PCGs of *A. amurensis* mitochondria typically begin with ATG start codons and preferentially end with A or T in their stop codon, which is similar to the codon preference of angiosperms [47]. The relative usage of the synonymous codons of *A. amurensis* and *O. sativa* was similar [48]. The results indicated a strong A/T bias in the third codon position of *A. amurensis* mitochondrial protein-coding genes, which is commonly observed in plant mitochondrial genomes [49]. SSRs are believed to play a major role in inducing the genetic variation underlying adaptation [50]. A connection between SSRs and stress resistance in plants has been reported. In *A. amurensis*, the highest proportions of SSRs in the nuclear genome and chloroplast genome were monomer nucleotide repeats and dimer nucleotide repeats [12], while tetrameric nucleotide repeats accounted for the highest proportion of SSRs in the mitochondrial genome. An increase in the number of nucleotide repeats in SSRs in the mitochondrial genome may promote the stress resistance of *Aquilegia* species during evolution [51].

### The phylogeny of *Aquilegia* based on mitochondrial genomes

In this study, we constructed phylogenetic trees based on SNPs of mitochondrial genomes to provide a new perspective on the phylogeny of *Aquilegia*. The results showed broadly similar phylogenetic trees based on nuclear or chloroplast genome data. The species of *Aquilegia* were roughly divided into two clades: the European-Asian clade and the North American clade, but the phylogenetic positions of some species were not completely consistent [11, 12, 52]. For example, phylogenetic trees based on mitochondrial and chloroplast genomes (unpublished) supported that *A. parviflora* was located in the European–Asian clade, while phylogenetic trees based on the nuclear genome (unpublished) showed that *A. parviflora* was closely related to North American species (Fig. 3). According to the phylogenetic tree of the mitochondrial genome, *A. yabeana* and *A. parviflora* were sister branches, while according to the phylogenetic tree of nuclear and chloroplast genomes (unpublished), *A. yabeana* was closely related to *A. rockii*, *A. oxysepala* var. *kansuensis* and *A. ecalcarata* (Fig. 3). Although both the chloroplast and mitochondrial genomes of *Aquilegia* were maternally inherited, they exhibited different phylogenetic relationships and differed from those of phylogenetic studies based on nuclear genomes. Therefore, the mitochondrial genome of plants could be an ideal molecular marker for phylogenetic studies, and an important tool for exploring biological evolution.

There were some inconsistencies in the phylogenetic trees of the mitochondrial, chloroplast and nuclear genomes [53, 54]. *Aquilegia* species were mainly cross-pollinated under natural conditions, characterized by extensive hybridization and high genetic diversity [55]. Some inconsistencies between the morphological and molecular phylogenies may indicate that hybridization played a major role in the evolution of the genus [56]. Organelle capture is the process by which the chloroplast genome of a plant infiltrates from one plant species to another after hybridization or backcrossing with the parental population. Organelle capture could occur frequently in species with sympatric distributions or contact zones and reproductive compatibility, which may explain the phylogenetic inconsistencies of *Aquilegia*. In addition, limited sampling, incomplete lineage classification, and differences in evolutionary rates could explain the phylogenetic reconstruction of the unclear and two organelle genomes [54, 57].

### Adaptative evolution of *Aquilegia*

*d*_*N*_*/d*_*S*_ analysis of the mitochondrial genomes of *Aquilegia* revealed that most of the genes were under negative selection during evolution, indicating that the protein-coding genes of the mitochondrial genome were well-conserved. The observed pattern of *d*_*N*_*/d*_*S*_ ratios is concordant with the expectations concerning the mitochondrial genomes for which negative selection was reported to be the predominant force of evolution [58]. This functional constraint of mitochondrial genes is associated with the important role of the organelle which is sustained by purifying selection, and is responsible for maintaining the long-term stability of biological structures by eliminating deleterious variations. As a consequence, protein-coding genes of mitochondrial genomes are conserved in land plants [59]. These protein-coding genes with *d*_*N*_*/d*_*S*_ <1 may play important roles in stabilizing the normal function of mitochondria. For example, the protein encoded by the gene *atp9* is one of the chains of the nonenzymatic membrane component (F0) of mitochondrial ATPase; the gene *nad2* is critical to the mitochondrial NADH dehydrogenase homologous subunits; and the gene *ccmC* may be involved in the export of heme to the mitochondrion for the biogenesis of c-type cytochromes [60–62].

However, the *d*_*N*_*/d*_*S*_ ratios of the genes *mttB*, *rpl5*, *rps4* and *rps13* were > 1, according to the selection pressure analysis of European-Asian species, indicating that these protein-coding genes were positively selected during evolution. The genes *rps4*, *rps12* and *rps13*, like the *rps14* gene, which was selected for all three comparisons, are involved in the small subunits of ribosomes. Moreover, they have different effects on the translation of mitochondrial mRNA. The *mttB* gene encodes a transport membrane protein, that is involved in the molecular functions of proton transmembrane transfer and proton-dynamic dependent protein transmembrane transfer functional protein and encodes a TATC-like protein (also known as orf X) [63]. Although little is known about the actual mitochondrial function of the *mttB* gene, this gene may be essential for mitochondrial function. The *mttB* gene was not lost in the mitochondrial genomes of 280 angiosperms species, which were intact [64]. The *mttB* gene controls the activity of trimethylamine methyltransferase to affect methylation, and is associated with metabolic alterations and oxidative stress [65]. For example, under high-salt conditions, the *mttB* gene was highly upregulated [66]. Transposable elements (TEs) in the plant mitochondrial genome can help plants adapt to the high-altitude environment, and the number of transposons around the *mttB* gene was greatest, which also indicated that the *mttB* gene was related to the adaptive evolution of plants [67]. The protein encoded by the *rpl5* gene is part of the 5 S ribonucleoprotein particle (5 S RNP), which is an important component of the ribosomal large subunit (LSU) and is required for rRNA [68]. The *rpl5* gene is a ribosomal protein-encoding gene with a high substitution rate and genetic variation in the organelle genome [69]. Previous studies have shown that mutations in large subunits of ribosomal protein genes inhibit sensitivity to temperature [70]. The *rpl5* gene was also positively selected during the evolution of *Paropyrum anemonoides* (Kar. & Kir.) Ulbr, and may be developing novel stress resistance mechanisms in Ranunculaceae plants [71]. The genes *mttB* and *rpl5* are highly sensitive to responses to stress and signaling molecules, indicating that these genes encode proteins that exhibit stress-ameliorating effects in addition to housekeeping [72]. Positive selection of these two genes involves fixing beneficial variation induced by environmental factors in a population and promoting the emergence of new phenotypes that can adapt to particular environmental conditions [73]. Adaptive evolution in European and Asian species may have been driven by a variety of environmental factors. In general, the genes *mttB* and *rpl5* might have developed novel functions related to stress resistance in European species and Asian species under positive selection pressure, enabling the species to be widely distributed [74].

## Conclusions

We successfully assembled and annotated the mitochondrial genome of *A. amurensis* and performed extensive analyses based on annotated genes. We obtained a circular chromosome and two linear chromosomes when repeated sequences were deleted, for a total length of 538,736 bp. We annotated 60 genes, including 33 protein-coding genes, 24 tRNA genes and 3 rRNA genes. Compared with the mitochondrial genome of model plants, the gene density of the mitochondrial genome of *A. amurensis* was lower. The phylogenetic tree of *Aquilegia* species based on mitochondrial genome showed similar topological structure to that of the nuclear and chloroplast genomes. The *Aquilegia* species were divided into two clades: the European-Asian clade and the North American clade, but some species had different phylogenetic positions. Finally, by analyzing selection pressure, we found that the mitochondrial genes *mttB* and *rpl5* may have contributed to the adaptive evolution of European and Asian species against adverse environments. This study complements the genetic knowledge available for the genus *Aquilegia* and provides new insights into the adaptive evolution of plants.

### Electronic supplementary material

Below is the link to the electronic supplementary material.

**Supplementary Material 1: Figure S1.** Mitochondrial genome sketch of *A. amurensis* (node ID marked in the figure). The orange color represents a major circular genome structure after resolving the duplicate region based on HiFi data

**Supplementary Material 2: Figure S2.** Phylogenetic relationships based on the mitochondrial genome of *Aquilegia*. The ML ultrafastbootstrap (ufbs) and BI posterior probability (PP) values are indicated above the branches. “*” are ufbs or PP of 100

**Supplementary Material 3: Table S1.** Information about the* Aquilegia* sequences data previously published. **Table S2.** Annotated genes list in the mitochondrial genome of *A. amurensis*. **Table S3.** SSRs in the mitochondrial genome of *A. amurensis*. **Table S4.** Tandem repeat sequences in the mitochondrial genome of *A. amurensis*

**Supplementary Material 4: Table S5.** Dispersed repeat sequences in the mitochondrial genome of *A. amurensis*

**Supplementary Material 5: Table S6.** dN/dS ratios of each gene in the mitochondrial genome of *Aquilegia*

**Supplementary Material 6: Table S7.** Nontandem repeat sequences in the mitochondrial genome of *A. amurensis*

## Data Availability

The *A. amurensis* mitochondrial genome sequence was deposited in the GenBank database (accession number OR818043, OR818044 and OR818045). Accession numbers for reference sequences downloaded from Genbank were provided in Table S2.

## Abbreviations

tRNA: Transfer RNA
rRNA: Ribosomal RNA
SNP: Single nucleotide polymorphism
SSR: Simple sequence repeat
CTAB: Cetyltrimethylammonium bromide
RSCU: Relative synonymous codon usage ratios
*d*_*N*_*/d*_*S*_: Ratios Nonsynonymous to synonymous substitution ratios
