# Supplementary figures and images for: Characterizing complete mitochondrial genome of Aquilegia amurensis and its evolutionary implications
Source: BMC Plant Biol. 2024 Feb 28;24:142. doi: 10.1186/s12870-024-04844-9 (PMC10900605; doi:10.1186/s12870-024-04844-9)

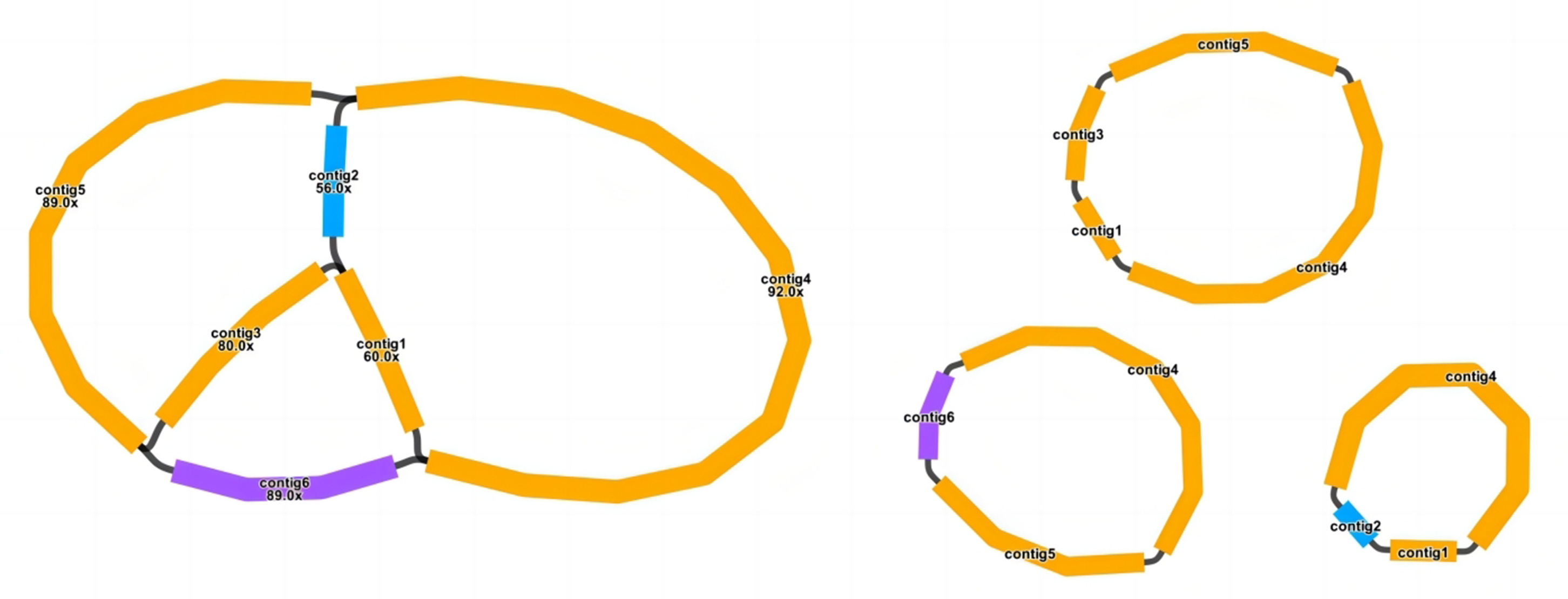

Supplement: Supplementary file 1 — Supplementary Material 1: Figure S1. Mitochondrial genome sketch of A. amurensis (node ID marked in the figure). The orange color represents a major circular genome structure after resolving the duplicate region based on HiFi data [file 12870_2024_4844_MOESM1_ESM.tif]

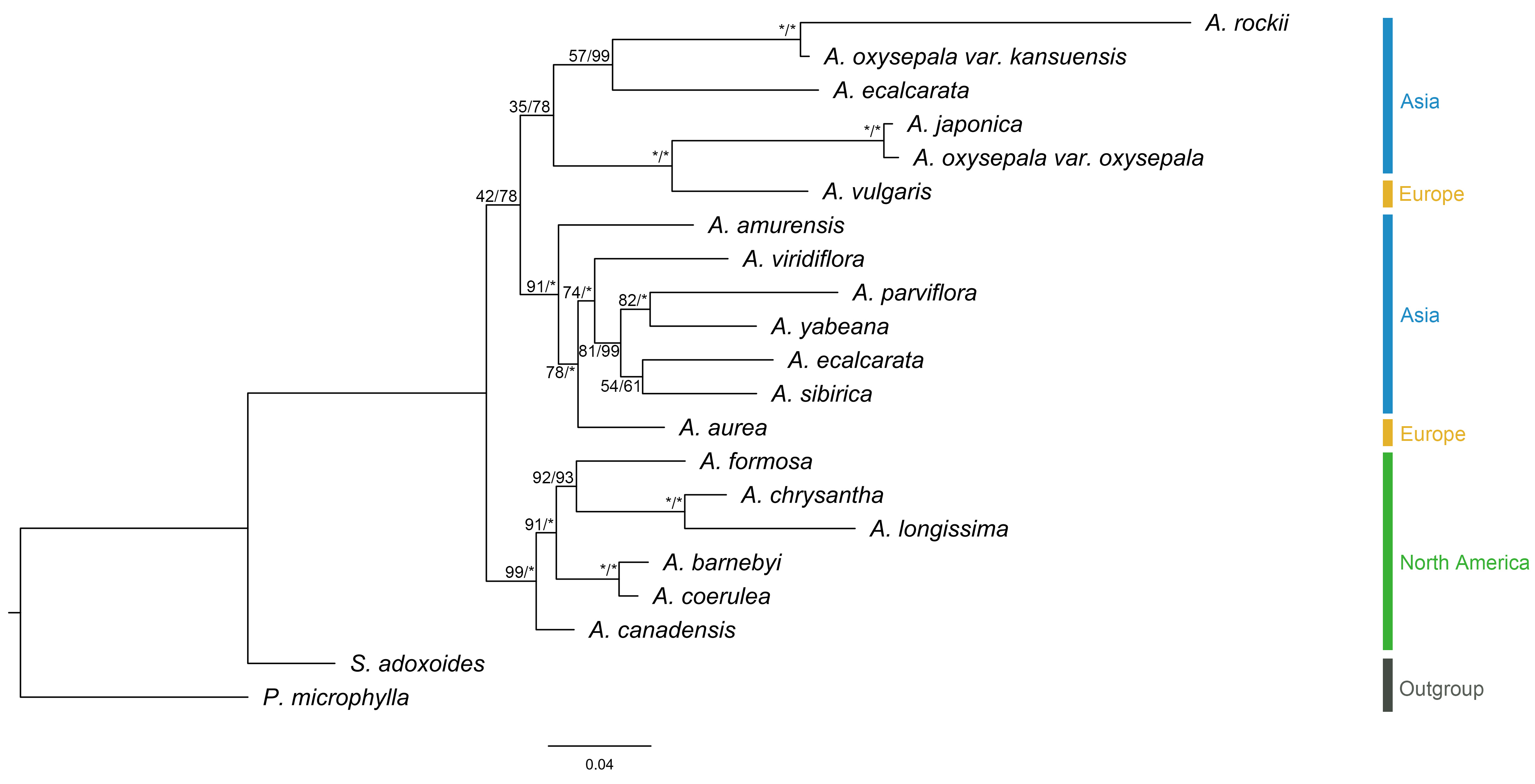

Supplement: Supplementary file 2 — Supplementary Material 2: Figure S2. Phylogenetic relationships based on the mitochondrial genome of Aquilegia. The ML ultrafastbootstrap (ufbs) and BI posterior probability (PP) values are indicated above the branches. “*” are ufbs or PP of 100 [file 12870_2024_4844_MOESM2_ESM.tif]
