# Supplementary material for: Characterizing complete mitochondrial genome of Aquilegia amurensis and its evolutionary implications
Source: BMC Plant Biol. 2024 Feb 28;24:142. doi: 10.1186/s12870-024-04844-9 (PMC10900605; doi:10.1186/s12870-024-04844-9)
Supplement: Supplementary file 3 — Supplementary Material 3: Table S1. Information about the Aquilegia sequences data previously published. Table S2. Annotated genes list in the mitochondrial genome of A. amurensis. Table S3. SSRs in the mitochondrial genome of A. amurensis. Table S4. Tandem repeat sequences in the mitochondrial genome of A. amurensis [file 12870_2024_4844_MOESM3_ESM.docx]

Table S1. Information about the *Aquilegia* sequences data previously published.

| Species | SRA no. | Distribution region | Reference |
| --- | --- | --- | --- |
| *A. sibirica* | SRR11508011 | Asia | Ballerin E S *et al*., 2020, Proceedings of the National Academy of Sciences of the United States of America |
| *A. ecalcarata* | SRR15649898 | Asia | Geng F D *et al*., 2022, Journal of Systematics and Evolution |
| *A. ecalcarata* | SRR15650159 | Asia |  |
| *A. parviflora* | SRS7452293 | Asia | Zhang W *et al*., 2023, Horticulture Research |
| *A. rockii* | SRS7452294 | Asia |  |
| *A. amurensis* | SRS9821777 | Asia |  |
| *A. viridiflora* | SRS9821766 | Asia |  |
| *A. yabeana* | SRS9821774 | Asia |  |
| 1. *oxysepala var.*   *kansuensis* | SRS9821776 | Asia |  |
| 1. *oxysepala var.*   *oxysepala* | SRS9821783 | Asia |  |
| *A. japonica* | SRS9821780 | Asia |  |
| *A. canadensis* | SRR892116 | North America | Filiault D *et al*., 2018, Elife |
| *A. coerulea* | SRR892117 | North America |  |
| *A. barnebyi* | SRR7965809 | North America |  |
| *A. longissima* | SRR7965810 | North America |  |
| *A. chrysantha* | SRR408559 | North America |  |
| *A. formosa* | SRR408554 | North America |  |
| *A. aurea* | SRR405095 | Europe |  |
| *A. vulgaris* | SRR404349 | Europe |  |

Table S2. Annotated genes list in the mitochondrial genome of *A. amurensis*.

| Group of genes | Gene name | Length | Start codon | Stop codon | Amino acid |
| --- | --- | --- | --- | --- | --- |
| ATP synthase | *atp1* | 1533 | ATG | TAA | 510 |
|  | *atp4* | 594 | ATG | TAA | 197 |
|  | *atp6* | 720 | ACG | CAA | 239 |
|  | *atp8* | 486 | ATG | TAA | 161 |
|  | *atp9* | 225 | ATG | TAA | 74 |
| NADH dehydrogenase | *nad1* | 978 | ACG | TAA | 324 |
|  | *nad2* | 1467 | ATG | TAA | 488 |
|  | *nad3* | 357 | ATG | TAA | 118 |
|  | *nad4* (×2) | 1488 | ATG | TGA | 495 |
|  | *nad4L* | 303 | ATG | TAA | 100 |
|  | *nad5* | 2013 | ATG | TAA | 670 |
|  | *nad6* | 618 | ATG | TAA | 205 |
|  | *nad7* | 1185 | ATG | TAG | 394 |
|  | *nad9* | 573 | ATG | TAA | 190 |
| Cytochrome c biogenesis | *cob* | 1182 | ATG | TGA | 393 |
| Ubiquinol cytochrome c reductase | *ccmB* | 621 | ATG | TGA | 206 |
|  | *ccmC* | 1050 | ATG | TAA | 349 |
|  | *ccmFC* | 1326 | ATG | CGA | 442 |
|  | *ccmFN* | 1734 | ATG | TGA | 577 |
| Cytochrome c oxidase | *cox1* | 1584 | ATG | TAA | 527 |
|  | *cox2* | 783 | ATG | TAA | 260 |
|  | *cox3* | 798 | ATG | TGA | 265 |
| Maturases | *matR* | 1980 | ATG | TAG | 659 |
| Transport membrane protein | *mttB* | 777 | CTT | TAG | 257 |
| Large subunit of ribosome | *rpl5* | 558 | ATG | TAA | 185 |
|  | *rpl10* | 489 | ATG | TAA | 162 |
|  | *rpl16* | 531 | ATG | TAA | 176 |
| Small subunit of ribosome | *rps4* | 1137 | TCC | TAG | 377 |
|  | *rps7* | 447 | ATG | TAA | 148 |
|  | *rps10* | 285 | ATG | CGA | 95 |
|  | *rps12* | 372 | ATG | TAG | 123 |
|  | *rps13* | 351 | ATG | TGA | 116 |
|  | *rps14* | 303 | ATG | TAG | 100 |
| Transfer RNAs | *trnA*-UGC | 73 | - | - | - |
|  | *trnC*-GCA | 73 | - | - | - |
|  | *trnD*-GUC (×2) | 71 | - | - | - |
|  | *trnD*-GUC | 74 | - | - | - |
|  | *trnE*-UCC (×2) | 72 | - | - | - |
|  | *trnF* | 73 | - | - | - |
|  | *Trn*-GCC (×2) | 72 | - | - | - |
|  | *TrnH* | 74 | - | - | - |
|  | *trnK*-UUU | 73 | - | - | - |
|  | *trnK*-UUU | 76 | - | - | - |
|  | *trnL* | 75 | - | - | - |
|  | *trnM*-CAU (×3) | 74 | - | - | - |
|  | *trnM-*CAU | 73 | - | - | - |
|  | *trnN*-GUU | 72 | - | - | - |
|  | *trnN*-GUU | 59 | - | - | - |
|  | *trnP*-UGG | 75 | - | - | - |
|  | *trnP*-CGG | 96 | - | - | - |
|  | *trnP*-CGG | 74 | - | - | - |
|  | *trnQ*-UUG | 65 | - | - | - |
|  | *trnQ*-UUG | 72 | - | - | - |
|  | *trnR*-UCG | 67 | - | - | - |
|  | *trnR*-UCU | 72 | - | - | - |
|  | *trnS*-UGA | 88 | - | - | - |
|  | *trnS*-GCU | 87 | - | - | - |
|  | *trnT* | 67 | - | - | - |
|  | *trnV* | 72 | - | - | - |
|  | *trnW*-CCA | 94 | - | - | - |
|  | *trnW*-CCA | 88 | - | - | - |
|  | *trnY* | 83 | - | - | - |
| Ribosomal RNAs | *rrn5* (×2) | 118 | - | - | - |
|  | *rrn18* | 2194 | - | - | - |
|  | *rrn26* | 3772 | - | - | - |

Notes: The numbers in parentheses represent the number of copies of the gene, such as (×2) means there are two copies.

Table S3 SSRs in the mitochondrial genome of *A. amurensis*

| Mitochondrial genome | SSR nr. | SSR type | SSR | size | start | end |
| --- | --- | --- | --- | --- | --- | --- |
| Chromosome 1 | 1 | p1 | (A)10 | 10 | 767 | 776 |
| Chromosome 1 | 2 | p2 | (TA)6 | 12 | 958 | 969 |
| Chromosome 1 | 3 | p1 | (T)11 | 11 | 1461 | 1471 |
| Chromosome 1 | 4 | p1 | (A)11 | 11 | 1626 | 1636 |
| Chromosome 1 | 5 | p3 | (AAT)5 | 15 | 1736 | 1750 |
| Chromosome 1 | 6 | p4 | (CATT)3 | 12 | 2807 | 2818 |
| Chromosome 1 | 7 | p1 | (A)11 | 11 | 3821 | 3831 |
| Chromosome 1 | 8 | p4 | (CTTT)3 | 12 | 6888 | 6899 |
| Chromosome 1 | 9 | p4 | (CTAT)3 | 12 | 13961 | 13972 |
| Chromosome 1 | 10 | p1 | (A)10 | 10 | 20547 | 20556 |
| Chromosome 1 | 11 | p4 | (CAGT)3 | 12 | 24453 | 24464 |
| Chromosome 1 | 12 | p4 | (GAAA)3 | 12 | 28081 | 28092 |
| Chromosome 1 | 13 | p1 | (A)10 | 10 | 28173 | 28182 |
| Chromosome 1 | 14 | p3 | (TTG)4 | 12 | 39353 | 39364 |
| Chromosome 1 | 15 | p4 | (TTGC)3 | 12 | 49763 | 49774 |
| Chromosome 1 | 16 | p4 | (TTTC)3 | 12 | 51494 | 51505 |
| Chromosome 1 | 17 | p2 | (CT)5 | 10 | 54068 | 54077 |
| Chromosome 1 | 18 | p4 | (TAGC)3 | 12 | 63992 | 64003 |
| Chromosome 1 | 19 | p3 | (AAG)4 | 12 | 67152 | 67163 |
| Chromosome 1 | 20 | p4 | (ATAG)3 | 12 | 72387 | 72398 |
| Chromosome 1 | 21 | p4 | (GGAA)3 | 12 | 79382 | 79393 |
| Chromosome 1 | 22 | p4 | (AAAG)3 | 12 | 80526 | 80537 |
| Chromosome 1 | 23 | p4 | (TTTG)3 | 12 | 80686 | 80697 |
| Chromosome 1 | 24 | p4 | (AAAG)3 | 12 | 83412 | 83423 |
| Chromosome 1 | 25 | p4 | (AGAA)3 | 12 | 85500 | 85511 |
| Chromosome 1 | 26 | p4 | (AAGG)3 | 12 | 103867 | 103878 |
| Chromosome 1 | 27 | p2 | (AG)5 | 10 | 108540 | 108549 |
| Chromosome 1 | 28 | p4 | (TTTC)3 | 12 | 126839 | 126850 |
| Chromosome 1 | 29 | p4 | (GGCG)3 | 12 | 129085 | 129096 |
| Chromosome 1 | 30 | p4 | (ATGG)3 | 12 | 131630 | 131641 |
| Chromosome 1 | 31 | p2 | (CT)5 | 10 | 134346 | 134355 |
| Chromosome 1 | 32 | p1 | (A)10 | 10 | 135576 | 135585 |
| Chromosome 1 | 33 | p6 | (ATAGAA)3 | 18 | 144195 | 144212 |
| Chromosome 1 | 34 | p4 | (CAGT)3 | 12 | 149941 | 149952 |
| Chromosome 1 | 35 | p2 | (GA)5 | 10 | 154299 | 154308 |
| Chromosome 1 | 36 | p4 | (ACTA)3 | 12 | 158418 | 158429 |
| Chromosome 1 | 37 | p4 | (TTCT)3 | 12 | 167471 | 167482 |
| Chromosome 1 | 38 | p3 | (CTT)4 | 12 | 176980 | 176991 |
| Chromosome 1 | 39 | p2 | (CT)5 | 10 | 181795 | 181804 |
| Chromosome 1 | 40 | p2 | (TA)5 | 10 | 185488 | 185497 |
| Chromosome 1 | 41 | p4 | (AAGA)3 | 12 | 186606 | 186617 |
| Chromosome 1 | 42 | p4 | (GAAT)3 | 12 | 190515 | 190526 |
| Chromosome 1 | 43 | p2 | (AG)5 | 10 | 198685 | 198694 |
| Chromosome 1 | 44 | p4 | (TTTC)3 | 12 | 202375 | 202386 |
| Chromosome 1 | 45 | p3 | (CTG)5 | 15 | 208337 | 208351 |
| Chromosome 1 | 46 | p5 | (CTATA)3 | 15 | 212490 | 212504 |
| Chromosome 1 | 47 | p1 | (A)12 | 12 | 219838 | 219849 |
| Chromosome 1 | 48 | p4 | (GCAA)3 | 12 | 221543 | 221554 |
| Chromosome 1 | 49 | p3 | (CTT)4 | 12 | 222730 | 222741 |
| Chromosome 1 | 50 | p2 | (TC)5 | 10 | 234310 | 234319 |
| Chromosome 1 | 51 | p4 | (GAAA)3 | 12 | 237532 | 237543 |
| Chromosome 1 | 52 | p4 | (CAAG)3 | 12 | 237554 | 237565 |
| Chromosome 1 | 53 | p5 | (TCAAG)3 | 15 | 237819 | 237833 |
| Chromosome 1 | 54 | p1 | (T)10 | 10 | 240810 | 240819 |
| Chromosome 1 | 55 | p1 | (A)11 | 11 | 248976 | 248986 |
| Chromosome 1 | 56 | p4 | (GAAA)3 | 12 | 249288 | 249299 |
| Chromosome 1 | 57 | p6 | (ATGTCG)3 | 18 | 251029 | 251046 |
| Chromosome 1 | 58 | p3 | (CAA)4 | 12 | 253722 | 253733 |
| Chromosome 1 | 59 | p1 | (A)10 | 10 | 254371 | 254380 |
| Chromosome 1 | 60 | p4 | (AGAC)3 | 12 | 259443 | 259454 |
| Chromosome 1 | 61 | p4 | (AGGA)3 | 12 | 262318 | 262329 |
| Chromosome 1 | 62 | p2 | (CT)5 | 10 | 263109 | 263118 |
| Chromosome 1 | 63 | p4 | (CCGA)3 | 12 | 263665 | 263676 |
| Chromosome 1 | 64 | p4 | (GCGA)3 | 12 | 265996 | 266007 |
| Chromosome 1 | 65 | p4 | (CCTG)3 | 12 | 271928 | 271939 |
| Chromosome 1 | 66 | p4 | (AAAG)3 | 12 | 271951 | 271962 |
| Chromosome 1 | 67 | p2 | (AT)6 | 12 | 272419 | 272430 |
| Chromosome 1 | 68 | p3 | (TTA)4 | 12 | 275753 | 275764 |
| Chromosome 1 | 69 | p3 | (ATA)4 | 12 | 276420 | 276431 |
| Chromosome 1 | 70 | p4 | (TCTA)3 | 12 | 281542 | 281553 |
| Chromosome 1 | 71 | p1 | (A)12 | 12 | 283197 | 283208 |
| Chromosome 1 | 72 | p1 | (T)10 | 10 | 295836 | 295845 |
| Chromosome 1 | 73 | p2 | (TA)5 | 10 | 298262 | 298271 |
| Chromosome 1 | 74 | p1 | (T)10 | 10 | 299001 | 299010 |
| Chromosome 1 | 75 | p1 | (A)10 | 10 | 303579 | 303588 |
| Chromosome 1 | 76 | p1 | (T)10 | 10 | 303827 | 303836 |
| Chromosome 1 | 77 | p3 | (TAA)4 | 12 | 307584 | 307595 |
| Chromosome 1 | 78 | p4 | (CTTA)3 | 12 | 308110 | 308121 |
| Chromosome 1 | 79 | p4 | (TATC)3 | 12 | 308843 | 308854 |
| Chromosome 1 | 80 | p4 | (TTGC)3 | 12 | 309095 | 309106 |
| Chromosome 1 | 81 | p4 | (TTGG)3 | 12 | 309168 | 309179 |
| Chromosome 1 | 82 | p4 | (TTGT)3 | 12 | 309701 | 309712 |
| Chromosome 1 | 83 | p4 | (GCTT)3 | 12 | 310532 | 310543 |
| Chromosome 1 | 84 | p1 | (T)10 | 10 | 310892 | 310901 |
| Chromosome 1 | 85 | p2 | (AG)5 | 10 | 312302 | 312311 |
| Chromosome 1 | 86 | p4 | (GTAA)3 | 12 | 314896 | 314907 |
| Chromosome 1 | 87 | p2 | (CT)5 | 10 | 318845 | 318854 |
| Chromosome 1 | 88 | p1 | (A)10 | 10 | 319161 | 319170 |
| Chromosome 1 | 89 | p4 | (ATGT)3 | 12 | 320250 | 320261 |
| Chromosome 1 | 90 | p1 | (A)14 | 14 | 321259 | 321272 |
| Chromosome 1 | 91 | p4 | (TTAT)3 | 12 | 326031 | 326042 |
| Chromosome 1 | 92 | p4 | (AAAG)3 | 12 | 330626 | 330637 |
| Chromosome 1 | 93 | p3 | (CTA)4 | 12 | 352567 | 352578 |
| Chromosome 1 | 94 | p3 | (AAC)4 | 12 | 355104 | 355115 |
| Chromosome 1 | 95 | p2 | (AG)5 | 10 | 356767 | 356776 |
| Chromosome 1 | 96 | p1 | (C)14 | 14 | 358338 | 358351 |
| Chromosome 1 | 97 | p4 | (TTTC)3 | 12 | 358599 | 358610 |
| Chromosome 1 | 98 | p2 | (CT)5 | 10 | 358973 | 358982 |
| Chromosome 1 | 99 | p1 | (T)10 | 10 | 359917 | 359926 |
| Chromosome 1 | 100 | p4 | (TCTA)3 | 12 | 375042 | 375053 |
| Chromosome 1 | 101 | p4 | (GCTA)3 | 12 | 382236 | 382247 |
| Chromosome 1 | 102 | p1 | (A)10 | 10 | 383119 | 383128 |
| Chromosome 1 | 103 | p2 | (TA)6 | 12 | 384868 | 384879 |
| Chromosome 1 | 104 | p4 | (AAAG)3 | 12 | 387090 | 387101 |
| Chromosome 1 | 105 | p2 | (TA)7 | 14 | 387218 | 387231 |
| Chromosome 1 | 106 | p1 | (T)10 | 10 | 387724 | 387733 |
| Chromosome 1 | 107 | p2 | (CT)5 | 10 | 388886 | 388895 |
| Chromosome 1 | 108 | p4 | (CTTA)3 | 12 | 389439 | 389450 |
| Chromosome 1 | 109 | p3 | (AGC)4 | 12 | 391413 | 391424 |
| Chromosome 1 | 110 | p4 | (AGAA)3 | 12 | 392922 | 392933 |
| Chromosome 1 | 111 | p4 | (ATGA)3 | 12 | 393090 | 393101 |
| Chromosome 1 | 112 | p1 | (A)10 | 10 | 393677 | 393686 |
| Chromosome 1 | 113 | p2 | (GT)5 | 10 | 395166 | 395175 |
| Chromosome 1 | 114 | p4 | (GCTA)3 | 12 | 395402 | 395413 |
| Chromosome 1 | 115 | p4 | (CTTT)3 | 12 | 396343 | 396354 |
| Chromosome 1 | 116 | p4 | (CACC)3 | 12 | 397144 | 397155 |
| Chromosome 1 | 117 | p3 | (AAG)4 | 12 | 397408 | 397419 |
| Chromosome 1 | 118 | p2 | (TA)7 | 14 | 399336 | 399349 |
| Chromosome 1 | 119 | p4 | (TTAT)3 | 12 | 401044 | 401055 |
| Chromosome 1 | 120 | p3 | (AAG)4 | 12 | 401554 | 401565 |
| Chromosome 1 | 121 | p2 | (AG)6 | 12 | 403587 | 403598 |
| Chromosome 1 | 122 | p4 | (ATCA)4 | 16 | 404433 | 404448 |
| Chromosome 1 | 123 | p2 | (TA)5 | 10 | 411515 | 411524 |
| Chromosome 1 | 124 | p2 | (GA)5 | 10 | 428654 | 428663 |
| Chromosome 1 | 125 | p1 | (A)12 | 12 | 429980 | 429991 |
| Chromosome 1 | 126 | p2 | (CA)5 | 10 | 431428 | 431437 |
| Chromosome 1 | 127 | p4 | (AGCT)3 | 12 | 433197 | 433208 |
| Chromosome 1 | 128 | p1 | (T)10 | 10 | 433420 | 433429 |
| Chromosome 1 | 129 | p4 | (GGAG)3 | 12 | 434811 | 434822 |
| Chromosome 1 | 130 | p4 | (TTCT)3 | 12 | 437051 | 437062 |
| Chromosome 1 | 131 | p4 | (AAGG)3 | 12 | 438376 | 438387 |
| Chromosome 1 | 132 | p2 | (AT)5 | 10 | 438520 | 438529 |
| Chromosome 1 | 133 | p3 | (AGT)4 | 12 | 447435 | 447446 |
| Chromosome 1 | 134 | p1 | (A)10 | 10 | 448781 | 448790 |
| Chromosome 1 | 135 | p4 | (AGCA)3 | 12 | 451121 | 451132 |
| Chromosome 1 | 136 | p4 | (AAAG)3 | 12 | 452196 | 452207 |
| Chromosome 1 | 137 | p4 | (AAAG)3 | 12 | 452252 | 452263 |
| Chromosome 1 | 138 | p2 | (AT)8 | 16 | 453238 | 453253 |
| Chromosome 1 | 139 | p4 | (GCAA)3 | 12 | 453485 | 453496 |
| Chromosome 1 | 140 | p4 | (CGCC)3 | 12 | 453755 | 453766 |
| Chromosome 1 | 141 | p3 | (CTA)4 | 12 | 454093 | 454104 |
| Chromosome 2 | 1 | p4 | (AAAG)3 | 12 | 7801 | 7812 |
| Chromosome 2 | 2 | p4 | (TTAT)3 | 12 | 11066 | 11077 |
| Chromosome 2 | 3 | p2 | (AT)5 | 10 | 15645 | 15654 |
| Chromosome 2 | 4 | p1 | (A)10 | 10 | 17467 | 17476 |
| Chromosome 2 | 5 | p4 | (CATA)3 | 12 | 18725 | 18736 |
| Chromosome 2 | 6 | p2 | (AG)5 | 10 | 22074 | 22083 |
| Chromosome 2 | 7 | p6 | (ACCTTC)3 | 18 | 23614 | 23631 |
| Chromosome 2 | 8 | p2 | (CT)5 | 10 | 28771 | 28780 |
| Chromosome 2 | 9 | p3 | (CTT)4 | 12 | 34776 | 34787 |
| Chromosome 2 | 10 | p2 | (CT)5 | 10 | 46783 | 46792 |
| Chromosome 3 | 1 | p4 | (AATG)3 | 12 | 13545 | 13556 |
| Chromosome 3 | 2 | p4 | (GCCG)3 | 12 | 17071 | 17082 |
| Chromosome 3 | 3 | p4 | (ACTG)3 | 12 | 20702 | 20713 |
| Chromosome 3 | 4 | p5 | (TGGAT)10 | 50 | 20924 | 20973 |
| Chromosome 3 | 5 | p4 | (AATC)3 | 12 | 24746 | 24757 |

Table S4 Tandem repeat sequences in the mitochondrial genome of *A. amurensis*

| Mitochondrial genome | Indices | Period | Copy | Consensus | Percent | Percent | Score | A | C | G | T | Entropy |
| --- | --- | --- | --- | --- | --- | --- | --- | --- | --- | --- | --- | --- |
|  |  | Size | Number | Size | Matches | Indels |  |  |  |  |  | (0-2) |
| Chromosome 1 | 3811--3871 | 29 | 2 | 30 | 93 | 6 | 106 | 68 | 8 | 13 | 9 | 1.38 |
| Chromosome 1 | 4535--4691 | 27 | 6 | 24 | 91 | 2 | 80 | 14 | 46 | 21 | 17 | 1.83 |
| Chromosome 1 | 4503--4583 | 42 | 1.9 | 42 | 97 | 0 | 155 | 12 | 46 | 19 | 20 | 1.82 |
| Chromosome 1 | 4559--4706 | 27 | 5.5 | 27 | 99 | 0 | 289 | 14 | 45 | 22 | 18 | 1.85 |
| Chromosome 1 | 35591--35640 | 24 | 2.1 | 24 | 96 | 0 | 93 | 20 | 22 | 4 | 54 | 1.61 |
| Chromosome 1 | 35850--35916 | 36 | 1.9 | 35 | 78 | 6 | 83 | 32 | 11 | 13 | 41 | 1.81 |
| Chromosome 1 | 66062--66093 | 16 | 2 | 16 | 87 | 0 | 50 | 28 | 21 | 15 | 34 | 1.94 |
| Chromosome 1 | 73578--73646 | 21 | 3.2 | 21 | 77 | 8 | 76 | 11 | 23 | 10 | 55 | 1.66 |
| Chromosome 1 | 91205--91249 | 20 | 2.3 | 19 | 78 | 14 | 51 | 33 | 15 | 40 | 11 | 1.83 |
| Chromosome 1 | 99977--100128 | 70 | 2.2 | 70 | 78 | 1 | 183 | 38 | 17 | 27 | 16 | 1.91 |
| Chromosome 1 | 108244--108273 | 15 | 2 | 15 | 93 | 0 | 53 | 33 | 10 | 30 | 26 | 1.89 |
| Chromosome 1 | 123122--123193 | 33 | 2.2 | 33 | 82 | 0 | 95 | 41 | 8 | 33 | 16 | 1.78 |
| Chromosome 1 | 136130--136213 | 33 | 2.5 | 33 | 94 | 0 | 147 | 13 | 40 | 11 | 34 | 1.81 |
| Chromosome 1 | 136243--136380 | 66 | 2.1 | 66 | 100 | 0 | 276 | 20 | 27 | 25 | 26 | 1.99 |
| Chromosome 1 | 158010--158050 | 20 | 2 | 20 | 90 | 0 | 68 | 31 | 26 | 26 | 14 | 1.95 |
| Chromosome 1 | 159128--159161 | 17 | 2 | 17 | 100 | 0 | 68 | 41 | 23 | 11 | 23 | 1.87 |
| Chromosome 1 | 214764--214851 | 45 | 2 | 45 | 81 | 0 | 120 | 13 | 22 | 47 | 15 | 1.81 |
| Chromosome 1 | 217584--217633 | 24 | 2.1 | 24 | 100 | 0 | 100 | 8 | 38 | 28 | 26 | 1.84 |
| Chromosome 1 | 223221--223358 | 72 | 1.9 | 72 | 86 | 0 | 213 | 23 | 23 | 21 | 31 | 1.98 |
| Chromosome 1 | 229142--229174 | 17 | 1.9 | 17 | 87 | 0 | 52 | 27 | 6 | 36 | 30 | 1.81 |
| Chromosome 1 | 233744--233802 | 29 | 2 | 29 | 83 | 0 | 83 | 35 | 28 | 18 | 16 | 1.93 |
| Chromosome 1 | 282054--282085 | 16 | 2 | 16 | 100 | 0 | 64 | 31 | 18 | 12 | 37 | 1.88 |
| Chromosome 1 | 296539--296617 | 39 | 2 | 39 | 100 | 0 | 158 | 22 | 27 | 17 | 31 | 1.97 |
| Chromosome 1 | 321155--321183 | 15 | 1.9 | 15 | 92 | 0 | 51 | 55 | 17 | 0 | 27 | 1.42 |
| Chromosome 1 | 323627--323656 | 13 | 2.3 | 13 | 100 | 0 | 60 | 30 | 13 | 0 | 56 | 1.37 |
| Chromosome 1 | 331769--331812 | 16 | 2.8 | 16 | 89 | 0 | 74 | 38 | 34 | 6 | 20 | 1.79 |
| Chromosome 1 | 396047--396100 | 27 | 2 | 27 | 100 | 0 | 108 | 33 | 11 | 14 | 40 | 1.82 |
| Chromosome 1 | 400049--400100 | 26 | 2 | 26 | 88 | 0 | 83 | 34 | 17 | 19 | 28 | 1.94 |
| Chromosome 1 | 400045--400110 | 26 | 2.3 | 29 | 72 | 17 | 74 | 28 | 27 | 15 | 28 | 1.96 |
| Chromosome 1 | 404048--404137 | 36 | 2.5 | 36 | 100 | 0 | 180 | 21 | 27 | 20 | 31 | 1.98 |
| Chromosome 1 | 424076--424237 | 72 | 2.3 | 71 | 92 | 1 | 273 | 35 | 16 | 22 | 25 | 1.95 |
| Chromosome 1 | 437718--437744 | 13 | 2.1 | 13 | 100 | 0 | 54 | 14 | 18 | 14 | 51 | 1.76 |
| Chromosome 1 | 442742--442781 | 20 | 2 | 20 | 80 | 0 | 52 | 12 | 17 | 25 | 45 | 1.83 |
| Chromosome 2 | 2566--2604 | 17 | 2.2 | 18 | 86 | 4 | 57 | 10 | 28 | 17 | 43 | 1.82 |
| Chromosome 2 | 9934--9971 | 19 | 2.1 | 18 | 85 | 9 | 53 | 68 | 5 | 18 | 7 | 1.34 |
| Chromosome 2 | 24246--24277 | 12 | 2.7 | 12 | 85 | 0 | 50 | 46 | 21 | 18 | 12 | 1.82 |
| Chromosome 2 | 24341--24392 | 24 | 2.1 | 25 | 82 | 3 | 69 | 53 | 17 | 21 | 7 | 1.68 |
| Chromosome 2 | 30665--30903 | 71 | 3.4 | 71 | 89 | 1 | 406 | 24 | 24 | 15 | 35 | 1.94 |
| Chromosome 3 | 3168--3196 | 15 | 1.9 | 15 | 92 | 0 | 51 | 37 | 27 | 6 | 27 | 1.82 |
